# Factors Influencing Background Parenchymal Enhancement in Contrast-Enhanced Mammography Images

**DOI:** 10.3390/diagnostics14192239

**Published:** 2024-10-08

**Authors:** Daniel Wessling, Simon Männlin, Ricarda Schwarz, Florian Hagen, Andreas Brendlin, Sebastian Gassenmaier, Heike Preibsch

**Affiliations:** 1 Department of Diagnostic and Interventional Radiology, University Hospital of Tuebingen, 72076 Tuebingen, Germany; simon.maennlin@med.uni-tuebingen.de (S.M.); ricarda.schwarz@med.uni-tuebingen.de (R.S.); andreas.brendlin@med.uni-tuebingen.de (A.B.); sebastian.gassenmaier@med.uni-tuebingen.de (S.G.); heike.preibsch@med.uni-tuebingen.de (H.P.); 2 Department of Diagnostic and Interventional Neuroradiology, University Hospital Heidelberg, Im Neuenheimer Feld 400, 69120 Heidelberg, Germany

**Keywords:** breast density, mammography, female

## Abstract

**Background:** The aim of this study is to evaluate the correlation between background parenchymal enhancement (BPE) and various patient-related and technical factors in recombined contrast-enhanced spectral mammography (CESM) images. **Material and Methods:** We assessed CESM images from 62 female patients who underwent CESM between May 2017 and October 2019, focusing on factors influencing BPE. A total of 235 images, all acquired using the same mammography machine, were analyzed. A region of interest (ROI) with a standard size of 0.75 to 1 cm^2^ was used to evaluate the minimal, maximal, and average pixel intensity enhancement. Additionally, the images were qualitatively assessed on a scale from 1 (minimal BPE) to 4 (marked BPE). We examined correlations with body mass index (BMI), age, hematocrit, hemoglobin levels, cardiovascular conditions, and the amount of pressure applied during the examination. **Results:** Our study identified a significant correlation between the amount of pressure applied during the examination and the BPE (Spearman’s ρ = 0.546). Additionally, a significant but weak correlation was observed between BPE and BMI (Spearman’s ρ = 0.421). No significant associations were found between BPE and menopausal status, cardiovascular preconditions, hematocrit, hemoglobin levels, breast density, or age. **Conclusions:** Patient-related and procedural factors significantly influence BPE in CESM images. Specifically, increased applied pressure and BMI are associated with higher BPE.

## 1. Background

Contrast-enhanced spectral mammography (CESM) was first introduced in the early 2000s. Therefore, it is a relatively recent advancement in breast imaging. It is an alternative to breast MRI and is particularly beneficial for patients with dense breast tissue, offering enhanced sensitivity compared to conventional mammography and ultrasound alone [1,2]. Since its introduction to the market, numerous studies have assessed the diagnostic accuracy of CESM, demonstrating a high sensitivity of approximately 90% and a commendable specificity of around 85% [3,4,5]. Before the examination, an iodine-based contrast agent is applied intravenously (1.5 mL/kg body weight). With a mammography device, two images are acquired during one round of compression—one image at a low energy level (26–33 kVp), and one at a high energy level (44–50 kVp). The low-energy image resembles a conventional digital mammogram. Using a complex recombination process with weighted logarithmic subtraction, a recombined image is produced, highlighting areas of increased contrast uptake. Pathological findings typically exhibit some degree of iodine uptake on CESM due to their neoangiogenesis [6,7,8].

CESM is performed without regard to the menstrual cycle to avoid delaying therapy in cases of suspicious findings. Consequently, some patients may exhibit benign parenchymal enhancement [4]. Elevated background parenchymal enhancement (BPE) in CESM images, like in breast MRI, can reduce the sensitivity of breast cancer detection [9]. Recent studies have introduced two approaches for analyzing background enhancement: a quantitative method involving the placement of a region of interest (ROI) in the CESM image to measure pixel intensity enhancement within a selected section [10,11] and a qualitative method used in routine clinical practice, where a radiologist evaluates the image on a numerical scale from 1 (minimal) to 4 (marked background enhancement) [12].

Currently, there are limited studies investigating the potential factors that influence BPE in CESM images. Similar to findings related to MRI, there are studies indicating that menstruation cycle timing could influence BPE in CESM images [13]. In addition, recent findings suggest that premenopausal status, high breast density, and younger age are associated with higher BPE [12,14]. However, the study situation remains unclear, as other studies investigating the same question found contradictory results [15].

Furthermore, a higher BPE might be associated with a higher risk of developing breast cancer, as implied by previous studies [15,16]. Similarly, studies on BPE in MRI found a higher risk of developing breast cancer in patient with a higher BPE [17,18]. Therefore, a comprehensive understanding of potential influencing factors and their possible interactions is essential.

Recent findings have demonstrated that the mammography machine can impact BPE in CESM images [19]. This suggests that operational and procedural factors during the mammogram could also affect BPE. However, to our knowledge, thus far no studies have examined these potential influencing factors.

In CT, MRI, and CESM, body mass index is a known influencing factor of tissue contrast, such that the volume of the applied contrast agent is adapted to the BMI [20]. In breast MRI, obesity is known to correlate with a higher BPE [21]. Thus, there might be a similar influence on BPE in CESM images.

Several potential influencing factors have not been investigated yet. In CT imaging, it is well known that cardiac preconditions can influence the timing of contrast agent admission [22]. This suggests that operational and procedural factors during mammography could also affect BPE. However, to our knowledge, thus far no studies have investigated these potential influencing factors. The potential effects on background enhancement in CESM, whose causes remain largely uninvestigated, are still unclear. As seen in abdominal CT imaging, the appropriate timing of contrast administration is potentially influenced by cardiac conditions such as a reduced left ventricular ejection fraction which could also affect soft tissue contrast [23].

Additionally, hematocrit and hemoglobin levels are parameters that are known to affect measurable blood flow, a relationship that is primarily examined in brain imaging [24,25]. The possible effects of these parameters on CESM images, which must be carefully timed to achieve optimal image quality, have yet to be determined.

The aim of this study is to evaluate the correlation between BPE and various patient-related and technical factors on recombined CESM.

## 2. Material and Methods

This is a retrospective observational study of patients who underwent CESM at a single center. This study was approved by the ethics committee for medical research of the University of Tuebingen (No. 159/2020 BO2). This study followed the regulations of the declaration of Helsinki.

### 2.1. Inclusion Criteria

From May 2016 to October 2019, a total of *n* = 65 patients who underwent clinically indicated CESM were retrospectively included in our study. No subjects were pregnant or lactating. Most of the patients (*n* = 47) underwent CESM because of suspicious findings on a mammography, ultrasound, or both (BI-RADS 4 or 5). In *n* = 15 cases, CESM was implemented because of an intensified follow-up after breast cancer therapy (*n* = 12) and within early detection programs for high-risk patients, who had contraindications for MRI examination and whose ultrasound findings were equivocal (*n* = 3). One patient had to be excluded because of implants, which might have influenced the quantitative results. Two patients had to be excluded because of missing clinical details. This resulted in a final total of *n* = 62 subjects.

### 2.2. Imaging Technique

All CESM images were acquired using Selenia^®^ Dimensions^®^ (Hologic, Marlborough, MA, USA) mammography machine. The patients received 1.5 mL/kg iodine-based contrast agents, injected intravenously with a flow rate of 3 mL per second. *n* = 43 patients received iopromid (Ultravist^®^ 300, Bayer, Leverkusen, Germany), while *n* = 19 patients were given iomerol (Imeron^®^ 350, Bracco, Milano, Italy). The first image was obtained 2 min after the application of the contrast agents; all images were acquired within less than 5 min. All examinations were performed by the same radiological technicians.

### 2.3. Image Analysis

**Figure 1:**
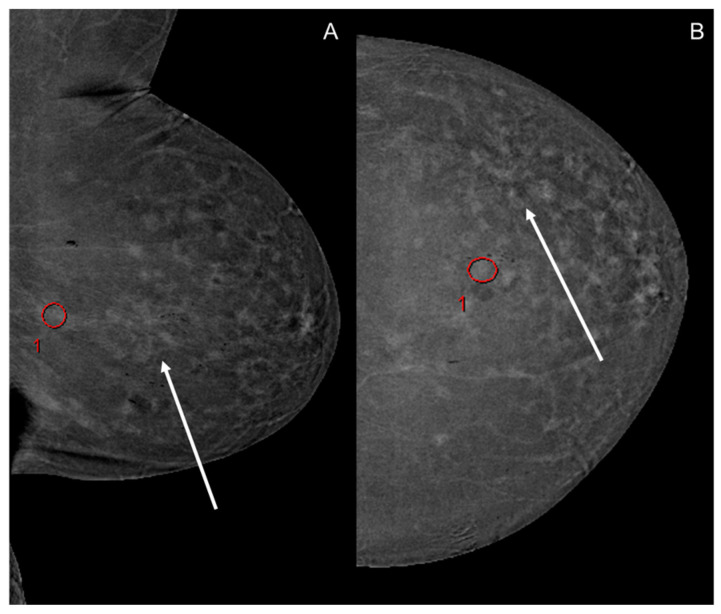
Example of qualitative BPE measurement using an oval-shaped ROI (red circles with numbering illustrating the number of ROIs within the image) in CESM images of the left breast of a 75-year-old woman in a mediolateral oblique (**A**) and craniocaudal projection (**B**). The contrast uptake of the histologically confirmed invasive carcinoma (arrows) can hardly be detected due to an extensive background enhancement.

For image evaluation, a dedicated workstation (Centricity RA1000, General Electric, Boston, MA, USA) was used. Image analysis was performed by setting an oval-shaped ROI of a size between 0.75 and 1.0 cm^2^ in the image sections with the highest BPE, whereby pathologic findings and known artifacts were not included. Within the ROI, a dimensionless value was measured and averaged, enabling the quantification of pixel intensity enhancement [13]. All images, which encompassed the two craniocaudal views as well as the two mediolateral views, were evaluated separately. Qualitative image analysis was performed by rating each view on a Likert scale from 1 (minimal BPE) to 4 (marked BPE) according to the CESM BI-RADS scoring system [26,27]. Quantitative and qualitative image analysis was performed independently by two radiologists with more than 3 years of experience in breast imaging after training with a senior radiologist with more than 10 years’ experience in breast imaging. Within the ROI, the maximum, minimum, average and standard deviation of the pixel intensity enhancement were determined. Breast density was determined for every patient (Figure 1).

### 2.4. Data Acquisition

All data were obtained from existing medical records. Patients with incomplete medical records were excluded from the final analysis, as stated above. The laboratory parameters were used only if the interval between the examination and the laboratory was no longer than 3 months. No postoperative laboratory parameters were used. Medical history data were used to identify cardiovascular preconditions that might lead to a higher BPE; hypertension, present coronary disease, heart insufficiency, cardiomyopathy, and ischemic heart disease were determined as cardiovascular preconditions. Menopausal status was divided into premenopausal and postmenopausal, whereas patients who were partaking in ongoing antihormonal therapy were categorized as postmenopausal.

### 2.5. Statistical Analysis

Statistical analysis was performed using MedCalc Statistical Software version 18.10 (MedCalc Software bvba, Ostend, Belgium; http://www.medcalc.org; 2018, accessed on 2 June 2022) and jmp (MP^®^, Version 15 SAS Institute Inc., Cary, NC, USA, 1989–2019). Data were tested for normal distribution using the Kolmogorov–Smirnov test.

In order to test the correlation between the first and the second reader, we used Spearman’s rank correlation test to determine the correlation of the quantitative data of both readers. The inter-reader correlation was then used to test for the correlation of the qualitative analysis. The strength of association was scaled with values ≤ 0 indicating no agreement, 0.01–0.30 indicating negligible agreement, 0.21–0.50 indicating weak agreement, 0.51–0.70 indicating moderate agreement, 0.71–0.90 indicating strong agreement, and 0.91–1.00 indicating perfect agreement [28].

First, we tested for correlation between our qualitative and quantitative results using Kendall’s τ. The strength of association was scaled as described above. For the correlation of the patient-dependent parameters, we averaged the pixel intensity enhancement of all images of one patient. Then, all numeric variables, including the applied pressure in Newtons, the hemoglobin (g/dL), hematocrit (%), and BMI (kg/m^2^), were tested for correlation with the qualitative and the quantitative BPE using Spearman’s rank correlation coefficient. The strength of association was scaled as described previously. For the correlation of the quantitative background enhancement and the categorical values (cardiac preconditions and menopausal status), a point biserial correlation was used. Ordinal data (breast density) and qualitative BPE were correlated using a Chi square test. To determine the strength of correlation, Spearman’s rank correlation test was used. For the comparison of the qualitative BPE, each image was correlated separately; Spearman´s correlation was used to determine the strength of the correlation. Ordinal parameters and the qualitative BPE were tested for correlation using Spearman´s rank correlation test.

## 3. Results

### 3.1. Image Analysis

We examined CESM images from a cohort of 62 patients with a median age of 61.29 (±13.81 years). Among these, five patients had only one breast examined, resulting in a total of two images per patient. In one instance, only two MLO (mediolateral oblique) images were obtained to minimize radiation exposure. Additionally, in one patient, two images were taken of one breast with a suspicious finding, while only one MLO view was acquired for the other breast, which was for radiation protection purposes. For the remaining 55 patients, 4 images per patient were obtained, leading to an overall analysis of 235 images.

### 3.2. Background Parenchymal Enhancement (BPE)

**Figure 2:**
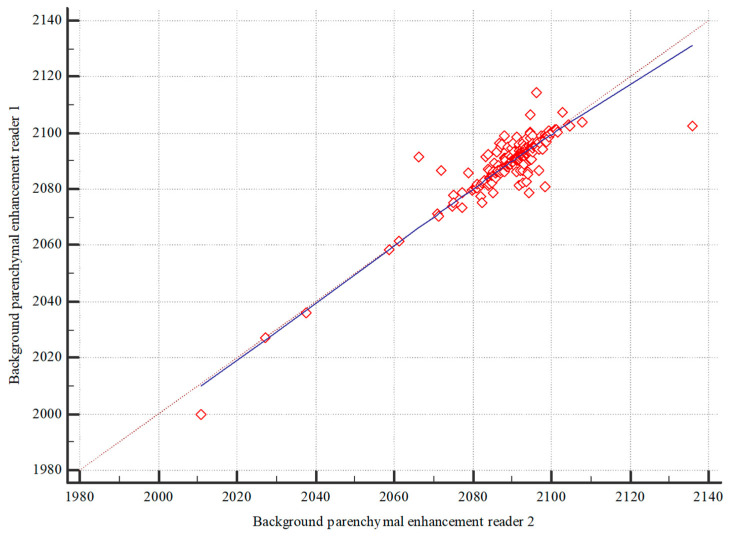
Comparison of the quantitative BPE of both readers.

**Figure 3:**
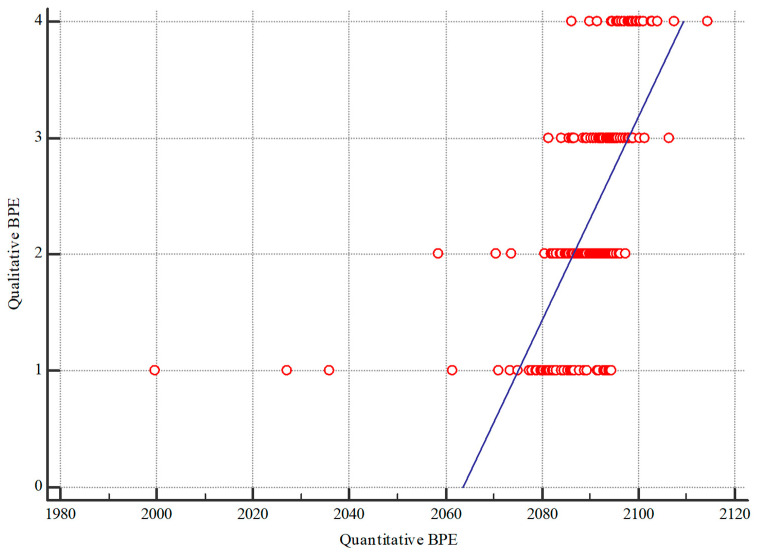
Scatter plot of the comparison of qualitative and quantitative BPE analysis.

The inter-reader reliability for the quantitative assessment of BPE demonstrated a strong correlation, with a Spearman’s correlation coefficient (ρ) of 0.804, as depicted in Figure 2. Similarly, the qualitative assessment showed a high intraclass correlation (ρ = 0.766). The first reader’s results are illustrated in Figure 3.

### 3.3. Quantitative BPE

We quantitatively analyzed 235 images, observing pixel intensity enhancements ranging from 1999.7 to 2114.3. The median quantitative BPE was revealed to be 2092, with an average of 2089. Detailed results are presented in Table 1.

### 3.4. Qualitative BPE

**Figure 4:**
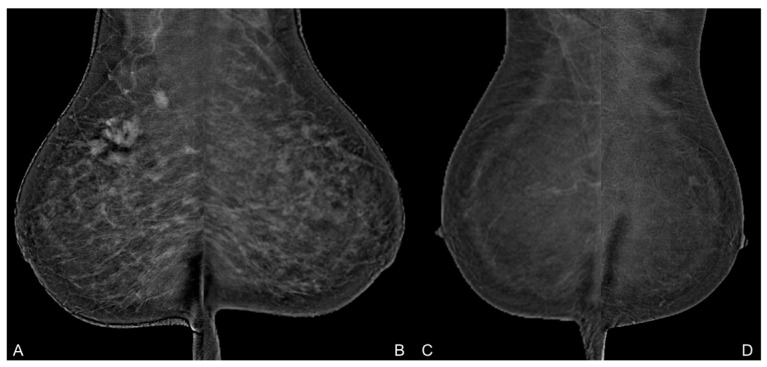
Comparison of the MLO projections of a 46-year-old premenopausal woman (**A**,**B**) and a 45-year-old premenopausal woman (**C**,**D**). While images (**A**,**B**) were acquired with a compression of 194 and 203 Newtons, a compression of 110 and 76 Newtons was used for the acquisition of images (**C**,**D**). BPE was rated qualitatively as 4 (marked) for image (**A**,**B**) and as 1 (minimal) for image (**C**,**D**).

The median qualitative BPE was 2, with an interquartile range (IQR) of 1–3, and the mean qualitative BPE was 2.299. As shown in Table 2, over 60% of the images were categorized as having mild or moderate BPE. A moderate and statistically significant correlation (τ = 0.529, *p* < 0.001) was observed between qualitative and quantitative analyses, as measured by Kendall’s τ. An example of the qualitative assessment of BPE is illustrated in Figure 4.

### 3.5. Hematologic Parameters

No statistically significant correlation was found between hematocrit and hemoglobin levels and quantitative BPE (*p* = 0.8213 and *p* = 0.456, respectively). Similarly, no significant association was observed between qualitative BPE and hematocrit (*p* = 0.328, Spearman’s ρ = 0.072) or hemoglobin (Spearman’s ρ = 0.199).

### 3.6. Cardiovascular Conditions

Of the 62 patients, 36 (60.57%) had pre-existing cardiovascular conditions, with data unavailable for 1 patient. No significant correlation between qualitative BPE and cardiovascular conditions was found (*p* = 0.282; r = 0.072). In addition, there was no significant correlation for the qualitative BPE (*p* = 0.530; Spearman’s τ = 0.042).

### 3.7. Breast Density

As illustrated in Table 3, most patients exhibited scattered (48.39%) or heterogeneously dense (38.71%) breast tissue. No statistically significant differences were found between groups with varying breast densities (χ^2^= 0.843). However, a statistically significant but negligible relationship was identified between breast density and qualitative BPE (*p* = 0.001, Spearman’s ρ = 0.214).

### 3.8. Body Mass Index (BMI) and Age

A statistically significant correlation (*p* = 0.004, Spearman’s ρ = 0.421) was observed between BMI and quantitative BPE. No correlation was found between BMI and qualitative BPE.

### 3.9. Age

Age showed no significant correlation with quantitative BPE (*p* = 0.566, Spearman’s ρ = −0.037) but a negligible negative correlation with qualitative BPE. Patient characteristics are illustrated in Table 3.

### 3.10. Applied Pressure

**Figure 5:**
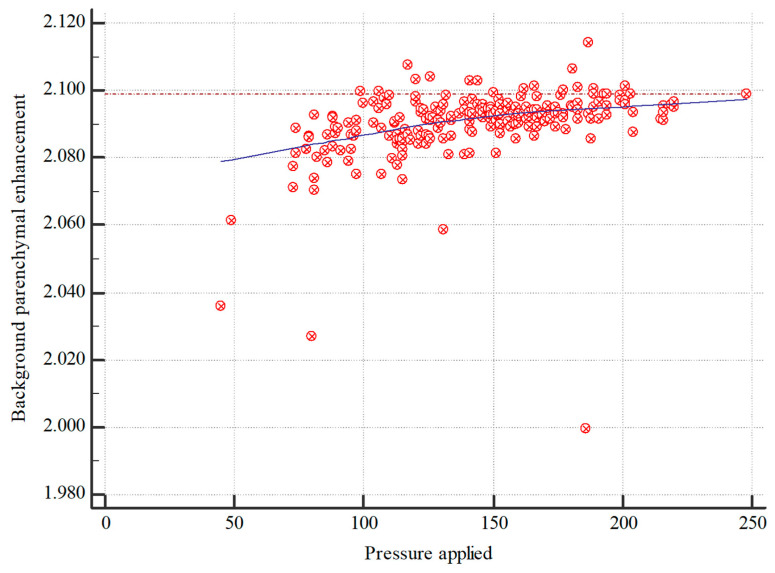
Correlation between applied pressure and quantitative BPE in CESM images.

The analysis revealed a significant (*p* < 0.001) and moderate (Spearman’s ρ = 0.546) correlation between applied pressure during CESM imaging and quantitative BPE, suggesting an approximately linear relationship (Figure 5). The correlation between applied pressure and qualitative BPE was also statistically significant (*p* < 0.001) but weaker (Pearson’s ρ = 0.273).

### 3.11. Menopausal Status

In our patient cohort, 42 patients were postmenopausal, 2 were undergoing antihormonal therapy and 18 were premenopausal. There was no statistically significant correlation between menopausal status and quantitative BPE (*p* = 0.562; r = 0.039). Accordingly, no significant correlation between qualitative BPE and menopausal status could be found (*p* = 0.490, Spearman’s τ = 0.0458).

## 4. Discussion

In this study, we analyzed the impact of procedural and patient-dependent factors on background parenchymal enhancement (BPE) in contrast-enhanced spectral mammography (CESM) images. Contrary to previous studies, our analysis did not show a statistically significant correlation between age or menopausal status and BPE within our study population. However, we identified a statistically significant correlation between the applied pressure during CESM imaging and BPE. This finding indicates that operating factors can influence BPE. To the best of our knowledge, this is the first study to investigate the impact of applied pressure on BPE. This novel insight accentuates the importance of considering procedural factors, such as the amount of pressure applied during imaging, as they can significantly affect the imaging outcomes. Further research is needed to verify these findings and investigate the underlying reasons through which applied pressure influences BPE.

In breast MRI, a higher amount of compression can impede the contrast uptake due to increased resistance within the intramammary blood vessels. Our study found that applying higher pressure during CESM leads to a higher amount of BPE. In CESM, the contrast agent is applied prior to clamping the breast in a mammography machine [3,29]. Therefore, there is enough time for the contrast agent to reach the intramammary blood vessels and the soft tissue. Due to the higher pressure, the tissue is compressed to a smaller volume, which may lead to overlays of the contrasted tissue that might lead to the impression of increased BPE. Furthermore, the pressure might cause extravasation of the contrast agent from the small venules to the soft tissue.

The significant impact of BMI on BPE could be explained by the correlation between the amount of pressure needed and the patient’s body mass index. Obese women, who tend to have larger breasts, often require higher pressure during imaging to obtain artifact-free images. Additionally, as obesity is a known precondition for cardiovascular diseases, this could further influence BPE. Therefore, it is reasonable to assume that multiple factors have an influence on background enhancement.

In accordance with previously published studies in MRI research, which demonstrated the influence of hematocrit on contrast dynamics, we investigated the influence of hematocrit and hemoglobin levels on BPE in CESM images [30]. Our analysis did not reveal any significant correlation. The contrast agent dosage in CESM imaging is adjusted according to the patient’s body weight, which may contribute to the lack of association.

Yu et al. found that a high measurable pixel intensity enhancement within a lesion could be essential in decision-making regarding the necessity of a biopsy. This suggests that while BPE itself may not be a risk factor, a notably higher pixel intensity enhancement within lesions provides valuable diagnostic information that could guide clinical interventions [11,31]. As only a few studies have investigated the factors influencing BPE, our findings emphasize the need for further verification of previously published findings. Some studies indicated a significant impact of age and breast density on BPE. However, our study did not find such correlations, suggesting that a larger cohort might be necessary to definitively determine the relationships between these factors and BPE. This highlights the importance of expanding research in this area to better understand the determinants of BPE and improve CESM imaging protocols.

Similar to findings related to MRI of the breast, a high BPE in CESM images can mask benign and malignant findings [9,32,33] and therefore reduce the sensitivity of the imaging process. Thus, it is essential to reduce BPE to a minimum. Studies have shown the potential influence of the menstrual cycle on BPE [13]. This would necessitate precise timing of the examination to minimize BPE. Consistent with other studies examining the impact of the menstrual cycle on BPE in MRI, our study found no significant correlation [34]. This suggests that larger cohort studies are required to validate the findings. Should the menstrual cycle have a lesser impact on BPE than anticipated, it would simplify the scheduling of examinations.

A synopsis of our evaluation with current studies suggests the influence of various factors on the background enhancement in CESM images, some of which are mutually dependent. With the pressure applied during mammography, we were able to identify a patient-independent influence factor. The results indicate that the performing mammographer, mostly a specially trained radiology technician, could directly influence the amount of BPE in the CESM images. In patients with otherwise high BPE due to other influencing factors, this would allow the mammographer to manually reduce the BPE and thereby improve the sensitivity. Furthermore, this could call other assumptions into question; for example, the assumption that BPE could be a potential risk factor for developing breast cancer [16].

Our study has some limitations. First of all, the study cohort was quite small; this limited the predictive power of the correlation between BPE and the patient-dependent factors, which should be tested in a more sophisticated study with a larger number of patients. Some of our patients had breast cancer therapy before the examination, which could impede the results. Regarding the cardiovascular preconditions, the pathophysiology of the included pathologies is known to be different. Thus, it would be necessary to distinguish these factors in a larger cohort to see whether there are differences between the different underlying diseases.

## 5. Conclusions

Higher pressure during contrast-enhanced mammography and a higher BMI correlate with a higher amount of background parenchymal enhancement in CESM images. Therefore, patient-related and operating factors influence the amount of BPE in CESM images. Age, menopausal status, breast density, hematologic parameters, and cardiovascular conditions did not correlate with BPE in CESM images.

## Figures and Tables

**Table 1:** Quartiles and distribution of the quantitative BPE.

100.0%	Maximum	2114.3
99.5%		2113.076
97.5%		2102.73
90.0%		2098.48
75.0%	Quartile	2094.8
50.0%	Median	2092
25.0%	Quartile	2086.6
10.0%		2081.36
2.5%		2069.59
0.5%		2004.632
0.0%	Minimum	1999.7

**Table 2:** Distribution of qualitative background enhancement.

BPE	*n*	%
1	46	19.83
2	105	44.30
3	52	21.94
4	32	13.92
All	235	

**Table 3:** Patient characteristics.

Age	*n*	%	ACR	*n*	%
20–30	0	0	A	2	3.23
30–40	4	6.45	B	30	48.39
40–50	9	14.52	C	24	38.71
50–60	17	27.42	D	6	9.68
60–70	11	17.74			
70–80	17	27.42			
80–90	4	6.45			
All	62				

Breast density according to classification of the American College of Radiology (ACR): A = almost entirely fatty tissue, B = Scattered areas of fibroglandular denisty, C = Heterogenously dense tissue, D = Extremely dense tissue.

## Data Availability

All data of the study is available from the responsible author on reasonable request.

## Abbreviations

BPE	Background parenchymal enhancement
BMI	Body mass index
CESM	Contrast-enhanced spectral mammography

## References

[1] James J.J., Tennant S.L. (2018). Contrast-enhanced spectral mammography (CESM). Clin. Radiol..

[2] Zhu X., Huang J.M., Zhang K., Xia L.J., Feng L., Yang P., Zhang M.Y., Xiao W., Lin H.X., Yu Y.H. (2018). Diagnostic Value of Contrast-Enhanced Spectral Mammography for Screening Breast Cancer: Systematic Review and Meta-analysis. Clin. Breast Cancer.

[3] Zanardo M., Cozzi A., Trimboli R.M., Labaj O., Monti C.B., Schiaffino S., Carbonaro L.A., Sardanelli F. (2019). Technique, protocols and adverse reactions for contrast-enhanced spectral mammography (CESM): A systematic review. Insights Imaging.

[4] Suter M.B., Pesapane F., Agazzi G.M., Gagliardi T., Nigro O., Bozzini A., Priolo F., Penco S., Cassano E., Chini C. (2020). Diagnostic accuracy of contrast-enhanced spectral mammography for breast lesions: A systematic review and meta-analysis. Breast.

[5] Mori M., Akashi-Tanaka S., Suzuki S., Daniels M.I., Watanabe C., Hirose M., Nakamura S. (2017). Diagnostic accuracy of contrast-enhanced spectral mammography in comparison to conventional full-field digital mammography in a population of women with dense breasts. Breast Cancer.

[6] Liu Y., Zhao S., Huang J., Zhang X., Qin Y., Zhong H., Yu J. (2020). Quantitative Analysis of Enhancement Intensity and Patterns on Contrast-enhanced Spectral Mammography. Sci. Rep..

[7] Schneider B.P., Miller K.D. (2005). Angiogenesis of breast cancer. J. Clin. Oncol..

[8] Lobbes M.B., Lalji U., Houwers J., Nijssen E.C., Nelemans P.J., van Roozendaal L., Smidt M.L., Heuts E., Wildberger J.E. (2014). Contrast-enhanced spectral mammography in patients referred from the breast cancer screening programme. Eur. Radiol..

[9] Travieso-Aja M.D.M., Naranjo-Santana P., Fernández-Ruiz C., Severino-Rondón W., Maldonado-Saluzzi D., Rodríguez Rodríguez M., Vega-Benítez V., Luzardo O.P. (2018). Factors affecting the precision of lesion sizing with contrast-enhanced spectral mammography. Clin. Radiol..

[10] Deng C.Y., Juan Y.H., Cheung Y.C., Lin Y.C., Lo Y.F., Lin G., Chen S.C., Ng S.H. (2018). Quantitative analysis of enhanced malignant and benign lesions on contrast-enhanced spectral mammography. Br. J. Radiol..

[11] Rudnicki W., Heinze S., Niemiec J., Kojs Z., Sas-Korczynska B., Hendrick E., Luczynska E. (2019). Correlation between quantitative assessment of contrast enhancement in contrast-enhanced spectral mammography (CESM) and histopathology-preliminary results. Eur. Radiol..

[12] Sorin V., Yagil Y., Shalmon A., Gotlieb M., Faermann R., Halshtok-Neiman O., Sklair-Levy M. (2020). Background Parenchymal Enhancement at Contrast-Enhanced Spectral Mammography (CESM) as a Breast Cancer Risk Factor. Acad. Radiol..

[13] Zhao S., Zhang X., Zhong H., Qin Y., Li Y., Song B., Huang J., Yu J. (2020). Background Parenchymal Enhancement on Contrast-Enhanced Spectral Mammography: Influence of Age, Breast Density, Menstruation Status, and Menstrual Cycle Timing. Sci. Rep..

[14] Karimi Z., Phillips J., Slanetz P., Lotfi P., Dialani V., Karimova J., Mehta T. (2021). Factors Associated With Background Parenchymal Enhancement on Contrast-Enhanced Mammography. AJR Am. J. Roentgenol..

[15] Savaridas S.L., Taylor D.B., Gunawardana D., Phillips M. (2017). Could parenchymal enhancement on contrast-enhanced spectral mammography (CESM) represent a new breast cancer risk factor? Correlation with known radiology risk factors. Clin. Radiol..

[16] Xu C., Jiang M., Lin F., Zhang K., Xie H., Lv W., Ji H., Mao N. (2023). Qualitative assessments of density and background parenchymal enhancement on contrast-enhanced spectral mammography associated with breast cancer risk in high-risk women. Br. J. Radiol..

[17] Watt G.P., Sung J., Morris E.A., Buys S.S., Bradbury A.R., Brooks J.D., Conant E.F., Weinstein S.P., Kontos D., Woods M. (2020). Association of breast cancer with MRI background parenchymal enhancement: The IMAGINE case-control study. Breast Cancer Res..

[18] Lee S.H., Jang M.J., Yoen H., Lee Y., Kim Y.S., Park A.R., Ha S.M., Kim S.Y., Chang J.M., Cho N. (2023). Background Parenchymal Enhancement at Postoperative Surveillance Breast MRI: Association with Future Second Breast Cancer Risk. Radiology.

[19] Wessling D., Männlin S., Schwarz R., Hagen F., Brendlin A., Olthof S.C., Hattermann V., Gassenmaier S., Herrmann J., Preibsch H. (2023). Background enhancement in contrast-enhanced spectral mammography (CESM): Are there qualitative and quantitative differences between imaging systems?. Eur. Radiol..

[20] Bae K.T., Seeck B.A., Hildebolt C.F., Tao C., Zhu F., Kanematsu M., Woodard P.K. (2008). Contrast enhancement in cardiovascular MDCT: Effect of body weight, height, body surface area, body mass index, and obesity. AJR Am. J. Roentgenol..

[21] Brown J.C., Ligibel J.A., Crane T.E., Kontos D., Yang S., Conant E.F., Mack J.A., Ahima R.S., Schmitz K.H. (2023). Obesity and metabolic dysfunction correlate with background parenchymal enhancement in premenopausal women. Obesity.

[22] Löbe S., Leuthäusser C., Pölkow A., Hilbert S., Sommer P., Bollmann A., Hindricks G., Paetsch I., Jahnke C. (2021). Optimal timing of contrast-enhanced three-dimensional magnetic resonance left atrial angiography before pulmonary vein ablation. Cardiol. J..

[23] Brink J.A. (2003). Contrast optimization and scan timing for single and multidetector-row computed tomography. J. Comput. Assist. Tomogr..

[24] van der Veen P.H., Muller M., Vincken K.L., Westerink J., Mali W.P., van der Graaf Y., Geerlings M.I. (2015). Hemoglobin, hematocrit, and changes in cerebral blood flow: The Second Manifestations of ARTerial disease-Magnetic Resonance study. Neurobiol. Aging.

[25] Thomas D.J., Marshall J., Russell R.W., Wetherley-Mein G., du Boulay G.H., Pearson T.C., Symon L., Zilkha E. (1977). Effect of haematocrit on cerebral blood-flow in man. Lancet.

[26] Meucci R., Pistolese C.A., Perretta T., Vanni G., Beninati E., Di Tosto F., Serio M.L., Caliandro A., Materazzo M., Pellicciaro M. (2022). Background Parenchymal Enhancement in Contrast-enhanced Spectral Mammography: A Retrospective Analysis and a Pictorial Review of Clinical Cases. In Vivo.

[27] Lee C., Phillips J., Sung J., Lewin J., Newell M. ACR BI-RADS^®^ ATLAS-MAMMOGRAPHY CONTRAST ENHANCED MAMMOGRAPHY (CEM). *A Supplement to ACR BI-RADS^®^ Mammography*
**2013**, *2022*. https://www.acr.org/-/media/ACR/Files/RADS/BI-RADS/BIRADS_CEM_2022.pdf.

[28] Akoglu H. (2018). User’s guide to correlation coefficients. Turk. J. Emerg. Med..

[29] Wong C.Y.Y., Lee S.Y.S., Mahmood R.D. (2024). Contrast-enhanced spectral mammography. Singap. Med. J..

[30] Sahoo P., Gupta P.K., Awasthi A., Pandey C.M., Patir R., Vaishya S., Saha I., Gupta R.K. (2016). Comparison of actual with default hematocrit value in dynamic contrast enhanced MR perfusion quantification in grading of human glioma. Magn. Reson. Imaging.

[31] Yu L., Wang Y., Xing D., Gong P., Chen Q., Lv Y. (2020). Background parenchymal enhancement on contrast-enhanced spectral mammography does not represent an influencing factor for breast cancer: A preliminary study. Medicine.

[32] Youn I., Choi S., Choi Y.J., Moon J.H., Park H.J., Ham S.Y., Park C.H., Kim E.Y., Kook S.H. (2019). Contrast enhanced digital mammography versus magnetic resonance imaging for accurate measurement of the size of breast cancer. Br. J. Radiol..

[33] Piccoli C.W. (1997). Contrast-enhanced breast MRI: Factors affecting sensitivity and specificity. Eur. Radiol..

[34] Dontchos B.N., Rahbar H., Partridge S.C., Lehman C.D., DeMartini W.B. (2019). Influence of Menstrual Cycle Timing on Screening Breast MRI Background Parenchymal Enhancement and Diagnostic Performance in Premenopausal Women. J. Breast Imaging.

